# Use of Evidence-Based Practices in Pregnancy and Childbirth: South East Asia Optimising Reproductive and Child Health in Developing Countries Project

**DOI:** 10.1371/journal.pone.0002646

**Published:** 2008-07-09

**Authors:** The SEA-ORCHID Study Group

**Affiliations:** Assiut University Hospital, Egypt

## Abstract

**Background:**

The burden of mortality and morbidity related to pregnancy and childbirth remains concentrated in developing countries. SEA-ORCHID (South East Asia Optimising Reproductive and Child Health In Developing countries) is evaluating whether a multifaceted intervention to strengthen capacity for research synthesis, evidence-based care and knowledge implementation improves adoption of best clinical practice recommendations leading to better health for mothers and babies. In this study we assessed current practices in perinatal health care in four South East Asian countries and determined whether they were aligned with best practice recommendations.

**Methodology/Principal Findings:**

We completed an audit of 9550 medical records of women and their 9665 infants at nine hospitals; two in each of Indonesia, Malaysia and The Philippines, and three in Thailand between January-December 2005. We compared actual clinical practices with best practice recommendations selected from the Cochrane Library and the World Health Organization Reproductive Health Library.

Evidence-based components of the active management of the third stage of labour and appropriately treating eclampsia with magnesium sulphate were universally practiced in all hospitals. Appropriate antibiotic prophylaxis for caesarean section, a beneficial form of care, was practiced in less than 5% of cases in most hospitals. Use of the unnecessary practices of enema in labour ranged from 1% to 61% and rates of episiotomy for vaginal birth ranged from 31% to 95%. Other appropriate practices were commonly performed to varying degrees between countries and also between hospitals within the same country.

**Conclusions/Significance:**

Whilst some perinatal health care practices audited were consistent with best available evidence, several were not. We conclude that recording of clinical practices should be an essential step to improve quality of care. Based on these findings, the SEA-ORCHID project team has been developing and implementing interventions aimed at increasing compliance with evidence-based clinical practice recommendations to improve perinatal practice in South East Asia.

## Introduction

The burden of mortality and morbidity related to pregnancy and childbirth remains concentrated in developing countries [1], [2]. This disparity continues with rates of neonatal mortality almost 10 times greater in South East Asia than developed regions [3], [4]. SEA-ORCHID, a five-year project, is evaluating whether a multifaceted intervention to strengthen capacity for research synthesis, evidence-based care and knowledge implementation improves adoption of best clinical practice recommendations and so leads to better health for mothers and babies [5]. This paper describes empirical evidence of current practices at the beginning of the SEA-ORCHID project for key aspects of maternal and perinatal health care in the participating countries.

## Methods

### Setting

This audit was conducted in nine hospitals across Indonesia, Malaysia, The Philippines and Thailand, with support from three sites in Australia. Different types of hospital were represented including tertiary referral hospitals (University and regional), provincial hospitals and district hospitals. All were selected as part of the SEA-ORCHID project.

Seven of the hospitals were tertiary referral institutions with regional referrals of women with a high risk pregnancy. Two hospitals were provincial or district institutions. Models of delivery care included a multidisciplinary approach with midwives (including nurses with midwifery qualifications) or obstetric specialists. All hospitals had obstetric specialists and caesarean section facilities available. Normal vaginal births were conducted by doctors and/or midwives (including nurses with midwifery qualifications) in all hospitals.

The SEA-ORCHID project settings and methods have been published elsewhere. [5]. The project was approved by the local ethics committee of each hospital and by the ethics committee of the administering institution in Australia (University of Sydney).

### Procedure

We reviewed the medical records of 9550 women (9665 infants including 111 twins and two sets of triplets) admitted to the labour wards at the nine study hospitals between January-December 2005. The duration of data collection varied according to the number of births per month at the participating hospital. Five hospitals in the sample collected data on a consecutive basis until reaching a total of at least 1000 women. To avoid potential biases associated with short data collection periods, the four largest hospitals sampled cases using various ratios to ensure there was at least a three-month minimum data collection period at each hospital.

Staff at each hospital were trained to prospectively audit medical records using pre-established data extraction forms. We extracted information about current maternal and perinatal practice according to beneficial forms of care and forms of care likely to be ineffective or harmful as suggested in the World Health Organization Reproductive Health Library No.7 [6] and the Cochrane Library [7] (Table 1). The primary caregiver at the birth provided details of the presence of maternal companionship during the labour.

In addition we collected information on maternal characteristics including age, parity, height and weight, and on birth outcomes including gestational age at birth, mode of birth, birth weight, Apgar scores and perinatal mortality.

**Table 1:** Recommended practices in maternal and perinatal health care

Recommended practice	Care practices assessed	Outcome intended to reduce
**Beneficial forms of care**		
Antibiotics for preterm prelabour rupture of membranes (pPROM) [15]	Use of antibiotics in women with preterm (<37 weeks) prelabour rupture of membranes	Chorioamnionitis; neonatal sepsis
Corticosteroids prior to preterm birth [16]	Use of antenatal corticosteroids in women at risk of preterm birth at <34 weeks gestation	Neonatal death; complications of preterm birth
Continuous support during labour [17]	Family member present with the woman during childbirth	Caesarean section rate
Magnesium sulphate for eclampsia and pre-eclampsia [18], [19], [20]	Use of magnesium sulphate for women with eclampsia and pre-eclampsia	Maternal death; eclampsia
Management of third stage of labour [21]	Use of uterotonic and controlled cord traction in the third stage of labour	Postpartum haemorrhage; maternal death
- appropriate administration of a prophylactic oxytocic at or after birth of the baby		
- controlled umbilical cord traction to deliver the placenta		
Intraoperative antibiotics during caesarean section [14]	Administration of a single dose of ampicillin or first generation cephalosporin after umbilical cord clamping at caesarean section	Maternal infection
Immunisation for Hepatitis B [22]	Vaccination of babies against Hepatitis B virus	Hepatitis B infection
**Forms of care likely to be unnecessary or harmful**		
Routine episiotomy [23]	Restrictive use of episiotomy	Perineal injury; maternal infection
Routine shaving* [24]	Avoidance of pubic hair shaving	Maternal infection
Routine enemas* [25]	Avoidance of use of enemas during labour	Maternal infection

* No clear evidence from Cochrane reviews to support or refute use, but identified as practices of importance to research and evaluate

Completed data extraction forms were sent from the hospitals to the project co-ordinating site in each country for manual data entry by the trained fieldworkers using a secure web-based database. The online form was set-up to minimise transcription errors by performing validation checks to detect discrepancies and missing data. A random sample of between 5 to 10% was independently checked by project staff at one of the Australian support sites to identify data processing errors.

### Data analysis

Descriptive analyses were performed across countries and between hospitals within countries. We used STATA software version 8.0 for data analysis [8]. We used frequencies to describe maternal characteristics, maternal and perinatal practices, as well as birth outcomes measured as categorical data. We used means and standard deviations to describe the continuous data variables of maternal age and gestational age at birth.

## Results

### Maternal characteristics and birth outcomes (Table 2)

For the 9550 mothers (and their 9665 babies) across the nine hospitals in South East Asia, the mean age per hospital ranged from 26 to 31 years. The rate of nulliparity varied, being over 40% in all hospitals of Indonesia, The Philippines and Thailand, and up to 64% in one Thai hospital but only 27% for one Malaysian hospital.

**Table 2:** Percent distribution of maternal and infant outcomes across nine hospitals in South East Asia

Countries	Indonesia			Malaysia			The Philippines			Thailand			
Hospitals	Overall	Tertiary	District	Overall	Tertiary 1	Tertiary 2	Overall	Tertiary 1	Tertiary 2	Overall	Regional	University	Provincial
Mothers^*^	**2086**	1019	1067	**2379**	1249	1130	**2085**	1026	1059	**3000**	1000	1000	1000
Infants^*^	**2113**	1028	1085	**2410**	1267	1143	**2098**	1026	1072	**3044**	1019	1013	1012
**Maternal age (years)** ***^σ^***	**30 (5.9)**	30 (6.0)	30 (5.7)	**30 (6.5)**	29 (6.4)	31(6.5)	**27 (6.6)**	26 (6.4)	28 (6.8)	**27 (6.1)**	26 (6.2)	28 (5.6)	27 (6.3)
**Nulliparous ^†^**	**46**	46	46	**31**	36	27	**48**	55	41	**58**	64	54	58
**Preterm birth (gestation <37 weeks) ^†^**	**10**	**14**	**6**	**10**	**10**	**10**	**7**	**6**	**9**	**11**	**15**	**10**	**8**
**Caesarean section ^†^**	**30**	29	31	**19**	22	17	**23**	12	33	**35**	34	34	39
**Low birth weight^ †^ (<2500 gm)**	**18**	20	16	**11**	11	11	**20**	19	20	**11**	15	9	10
**APGAR Scores <7 at 5 minutes^†^**	**8**	10	6	**1**	1	1	**1**	1	2	**2**	2	2	2
Perinatal death^*^ Per 1000 total births	**35**	42	29	**12**	11	12	**4**	5	3	**16**	15	21	13

Figures are number^*^ and mean (standard deviation)^σ^ or percentage^ †^ (rounded to the nearest whole number)

For the 9665 babies, the preterm birth rate (<37 weeks gestation) ranged from 6% to 15%. Overall 30% of all babies were born by caesarean section but the rate varied considerably across the nine hospitals ranging from 12% to 39%. Around a fifth of babies were born by caesarean section in the Malaysian hospitals and over a third in the Thai hospitals. Overall, 18% of babies were of low birth weight (<2500g) with rates ranging from 9% to 20%. Rates of babies with Apgar scores less than seven at five minutes and perinatal death were highest in the two Indonesian hospitals, compared with rates in the other hospitals. Stillbirths accounted for more than 60% of the perinatal deaths in most hospitals across the countries. There were no maternal deaths prior to discharge reported in any of the hospitals during the audit.

The variation in rates between countries and between hospitals within the same country are likely to be influenced by the sociodemographics and risk status of the populations they care for, and the type of hospital (tertiary, provincial or district).

### Beneficial forms of care

The recommended beneficial clinical practices were classified according to periods of pregnancy care: antenatal, intrapartum and postpartum (Table 3). The use of these clinical practices varied markedly across the nine hospitals.

**Table 3:** Percentage distribution of practices of beneficial forms of care

Care Practices	Indonesia			Malaysia			The Philippines			Thailand			
	Overall	Tertiary	District	Overall	Tertiary 1	Tertiary 2	Overall	Tertiary 1	Tertiary 2	Overall	Regional	University	Provincial
***Antenatal period***													
**MgSO_4_ for eclamptic fit**	**100**	100	100	**100**	100	-	**83**	100	80	**100**	100	-	-
**Antibiotic use for pPROM**	**91**	88	100	**62**	83	44	**92**	99	67	**73**	91	65	63
**MgSO_4_ for pre-eclampsia**	**100**	100	100	**24**	44	13	**100**	91	100	**64**	57	82	67
**Corticosteroids given to women who gave birth at <34 weeks**	**9**	9	10	**55**	59	47	**15**	0	21	**73**	86	60	60
***Intrapartum period***													
**Management of the third stage of labour**													
**Appropriate prophylactic oxytocic given during third stage**	**26**	26	26	**5**	10	1	**27**	<1	64	**58**	4	76	98
**Controlled cord traction to deliver the placenta**	**100**	100	100	**100**	100	99	**98**	100	96	**56**	31	43	96
**Family support during labour**	**41**	38	45	**61**	86	32	**10**	<1	19	**53**	83	31	44
**Appropriate antibiotic use for caesarean section**	**0**	0	0	**<1**	<1	0	**5**	0	7	**49**	72	82	<1
***Postpartum period***													
**Immunisation for Hepatitis B**	**2**	0	3	**99**	99	99	**2**	<1	4	**99**	99	98	99

pPROM: preterm prelabour rupture of membranes, MgS0_4_ : Magnesium Sulphate

Figures are percentages (rounded to the nearest whole number)

#### Care practices during the antenatal period to treat pregnancy complications (Table 3)

Women with pre-eclampsia represented 4% of the sample; with nearly three-quarters of the women with pre-eclampsia receiving magnesium sulphate. The rate of practice of this intervention varied among the hospitals within Malaysia (range 13% to 44%) and Thailand (range 57% to 82%) but was 100% in both Indonesian hospitals. There were 15 (0.16%) cases of eclampsia. Magnesium sulphate was used in all 15 (100%) of these cases of eclampsia, and in all countries involved in the audit.

Women with preterm prelabour rupture of the membranes (pPROM) received antibiotics 80% of the time. Although this practice was observed in all hospitals, the rates of administration varied across countries (range 62% to 92%) and hospitals (range 44% to 100%). In Indonesia, use was appropriately high whereas variation of the practices was seen among hospitals within each country.

Antenatal corticosteroid administration to women with preterm birth at <34 weeks was infrequently used in most hospitals compared with other clinical practices in the antenatal period. The highest rate of antenatal corticosteroid use was 86% in a regional hospital in Thailand. In the Malaysian hospitals about half the women who gave birth at <34 weeks had been given antenatal corticosteroids. In the Indonesian hospitals this dropped to around 10% and in one tertiary hospital in The Philippines no administration of corticosteroids was recorded. Use of repeat courses of antenatal corticosteroids was less than 15% across all hospitals included in the audit.

#### Care practices during the intrapartum period (Table 3)

Companionship in labour was assessed as to whether the woman had a family member present during childbirth. Rates for family support during labour varied widely, ranging from <1% at a Philippine hospital to 86% at a Malaysian hospital. Among the Malaysian hospitals the rates differed from 32% to 86%. In Thailand the rates of family support during labour were 31% at the university hospital, 44% at the provincial hospital and 83% at the regional hospital.

There were wide variations in the administration of an appropriate prophylactic oxytocic during third stage of labour both between countries and within countries. Rates for the administration of a uterotonic varied from <1% at one Philippine hospital to 98% at the Thai provincial hospital. Among the Thai hospitals rates of the practice were 4%, 76% and 98%. The variation was similar in The Philippines with one hospital giving oxytocics in two-thirds of cases and <1% in the other.

Controlled umbilical cord traction to deliver the placenta was routinely performed in the Indonesian, Malaysian and Philippine hospitals. In Thailand, the rates were below 50% in the regional and tertiary hospitals but 96% in the provincial hospital.

Use of antibiotic prophylaxis for caesarean section in a single does administered after clamping of the umbilical cord was mostly very low or non-existent across all the hospitals. About a quarter of women who gave birth by caesarean were given appropriate antibiotic prophylaxis (534 out of 2564), over 95% of these women were from two Thai hospitals.

Although antibiotics were often given in other hospitals, the timing of administration was preoperatively or postoperatively, and multiple doses were often prescribed.

#### Practices during postpartum period (Table 3)

Immunising babies against the hepatitis B virus was the only postpartum neonatal care practice assessed in our project. This intervention was only routinely practiced in the Malaysian and Thai hospitals.

### Forms of care likely to be ineffective or harmful (Table 4)

Episiotomy was the form of care likely to be harmful which was most frequently practiced across all the hospitals and in all four countries. The overall episiotomy rate for women giving birth vaginally was 65%. Episiotomies were liberally performed in all three hospitals in Thailand, and in only one Malaysian hospital did the rate drop below one-third. Pubic hair shaving, another ineffective and potentially harmful form of care, was commonly practiced in all countries with rates as high as 98% in one Thai hospital. In only one Philippine hospital was this observed as an occasional practice. Use of enemas during labour, an ineffective practice, was occasionally used in four hospitals across all countries and was highest in one Thai (61%) and one Malaysian (44%) hospital.

**Table 4:** Percentage distribution of practices of forms of care likely to be harmful

Care Practices	Indonesia			Malaysia			The Philippines			Thailand			
	Overall	Tertiary	District	Overall	Tertiary 1	Tertiary 2	Overall	Tertiary 1	Tertiary 2	Overall	Regional	University	Provincial
**Episiotomy for vaginal births**	**54**	49	60	**47**	61	31	**64**	64	64	**91**	95	93	86
**Pubic hair shaving**	**28**	26	29	**33**	24	42	**26**	12	40	**74**	43	79	98
**Enema use**	**17**	17	18	**22**	2	44	**1**	<1	3	**30**	1	29	61

Figures are percentages (rounded to the nearest whole number).

## Discussion

This study describes the rates of use of selected practices of key aspects of perinatal care reported in the medical records of nine hospitals in Indonesia, Malaysia, The Philippines and Thailand. The findings show high rates of compliance for some evidence-based recommendations for perinatal care and wide divergence for others. Practices that were in line with recommendations across most hospitals for the beneficial forms of care were controlled cord traction, one of the components of the active management of the third stage of labour, and treating eclampsia with magnesium sulphate. The unnecessary practice of enema use was appropriately widely avoided. The highest level of divergence from best practice recommendations in most countries was not administering appropriate antibiotic prophylaxis for caesarean section. Liberal use of episiotomy for women having a vaginal birth is not recommended but was often inappropriately practiced across the hospitals in all four countries, demonstrating lack of adoption of the evidence-based recommendation of restrictive episiotomy. Other forms of perinatal care such as pubic hair shaving and the use of enemas during labour varied in rates of compliance across all countries and even between hospitals within the same country.

The principles of evidence-based practice are to encourage health professionals to use practices with proven benefit and eliminate the use of those shown to be ineffective or harmful. Effective implementation of beneficial practices in developing regions [9], [10], such as South East Asia, should lead to a reduction in maternal and neonatal mortality and morbidities.

Our findings are consistent with three previous reports of perinatal practice from the Asian and Arab world [11]–[13]. The first was reported by the Choices and Challenges in Changing Childbirth Research Network [11]. The network documented routine obstetric practices for normal labour and birth in Egypt, Lebanon, Syria and the West Bank, and compared these with evidence-based recommendations. They showed the practices for normal labour were largely not in accordance with the World Health Organization evidence-based classification of practices for normal birth. The second report described facility-based practices for normal labour and birth [12]. Forty-four clinical practices observed in a busy Egyptian teaching hospital were categorised according to World Health Organization Technical Working Group on normal birth classification of normal birth practices. This study concluded that practices for normal labour were largely not consistent with the World Health Organization evidence-based classification of practices for normal births. The third report compared practices of selected childbirth care procedures against evidence-based information and explored user and provider views about each procedure in four hospitals in Shanghai, China [13]. They concluded that obstetric practices of the hospitals studied were not following best available evidence.

There is clear evidence of benefit to perinatal health outcomes with use of appropriate antibiotic prophylaxis for caesarean section, use of antenatal corticosteroids for women at risk of preterm birth and family support during labour. Our findings however, show that these clinical practices were rarely performed in most of the included hospitals, with high rates of variation across the countries. It is likely that there are a range of barriers to all these clinical practices in our study settings, but detailed exploration of these was outside the scope of this initial audit. Although we were not able to interview the care providers or directly observe the clinical practices at the time of this survey, these were planned for a later stage of this project.

Recommendation of use of appropriate antibiotic prophylaxis for caesarean section is defined as administration of a single dose of ampicillin or first generation cephalosporin after umbilical cord clamping of the baby [14]. We found that most non-concordant practices relating to antibiotic prophylaxis for caesarean section arose from giving multiple doses or administering antibiotics pre or post operatively. In some clinical settings multiple doses of antibiotics at pre-operative or post-operative times may be appropriate.

One advantage of our study was that the prospective, sequential collection of data provided accurate information of individual pregnant women and their babies, directly extracted from the medical records of individual pregnant women rather than derived from interviewing health care providers. In addition, the information was extracted prospectively and therefore the findings closely reflect the actual practices of perinatal health care in the nine hospitals of the four South East Asian countries. A limitation of such data collection is that there might be some interventions that were practiced but not well documented in medical records. However, this is likely to be minimal as most information in medical records of the hospitals involved in the study is standardised for the care practices considered.

In summary, few practices of perinatal health care in the nine hospitals within the four South East Asian countries were consistent with best available evidence from Cochrane reviews and the World Health Organization Reproductive Health Library recommendations. At the individual hospitals the audit results were used to evaluate the barriers to adoption of appropriate practices and elimination of inappropriate practices followed by strategies to increase the use of evidence-based practices in perinatal health care. The recording of clinical practices within this audit was a pre-requisite to identifying problems to be addressed.

Based on our findings, the SEA-ORCHID project team developed and implemented interventions that aimed to build capacity for research conduct, synthesis and translation that would increase compliance with evidence-based clinical practice recommendations and so improve maternal and perinatal care.
