# Correction: Use of Evidence-Based Practices in Pregnancy and Childbirth: South East Asia Optimising Reproductive and Child Health in Developing Countries Project

**DOI:** 10.1371/annotation/f3fa6c7e-2d8b-423b-824b-0373dee3c86c

**Published:** 2008-07-23

**Authors:** The SEA-ORCHID Study Group

The Author Contributions section incorrectly assigns a single member of the SEA-ORCHID Study Group to the study’s design, conduct, analysis, interpretation, and reporting. The correct contribution of each member of the SEA-ORCHID Study Group is listed in the Acknowledgments section. 

